# The Effect of Different White Spot Lesion Treatments on the Enamel Microhardness—An In Vitro Pilot Study

**DOI:** 10.3390/dj13110496

**Published:** 2025-10-27

**Authors:** Milena Milanović, Miloš Beloica, Zoran Mandinić, Jelena Juloski, Miloš Petrović, Dušan Kosanović, Miloš Todorović, Maja Dimitrijević, Aleksandar Jakovljević, Miloš Vorkapić, Dragan Stanimirović

**Affiliations:** 1 Clinic for Pediatric and Preventive Dentistry, School of Dental Medicine, University of Belgrade, 11000 Belgrade, Serbia; milena.milanovic@stomf.bg.ac.rs (M.M.); zoran.mandinic@stomf.bg.ac.rs (Z.M.); jelena.juloski@stomf.bg.ac.rs (J.J.); dusan.kosanovic@stomf.bg.ac.rs (D.K.); milos.todorovic@stomf.bg.ac.rs (M.T.); maja.dimitrijevic@stomf.bg.ac.rs (M.D.); 2 Faculty of Technology and Metallurgy, University of Belgrade, 11000 Belgrade, Serbia; mpetrovic@tmf.bg.ac.rs; 3 Department of Pathophysiology, School of Dental Medicine, University of Belgrade, 11000 Belgrade, Serbia; a.jakovljevic@stomf.bg.ac.rs; 4 Scientific Laboratories, Implant-Research Center, School of Dental Medicine, University of Belgrade, 11000 Belgrade, Serbia; 5 Institute of Chemistry, Technology and Metallurgy, National Institute of the Republic of Serbia, University of Belgrade, 11000 Belgrade, Serbia; worcky@nanosys.ihtm.bg.ac.rs; 6 Clinic for Periodontology and Oral Medicine, School of Dental Medicine, University of Belgrade; 11000 Belgrade, Serbia; dragan.stanimirovic@stomf.bg.ac.rs

**Keywords:** white spot lesion, minimally invasive dentistry, enamel microhardness, resin infiltration, fluoride varnish, CPP-ACP

## Abstract

**Background/Objectives:** Dental caries, one of the most common oral diseases worldwide, represents a major public health concern. Contemporary dentistry has established several non-invasive approaches and resin infiltration, as a micro-invasive path, in the treatment of white spot lesions (WSLs). This study aimed to evaluate the effect of different WSL treatments on enamel surface microhardness. **Materials and Methods:** Seventy-five intact human premolars extracted upon orthodontic indication and the demineralizing solution composed of acetic acid, monopotassium phosphate and calcium chloride with pH = 4.4 and exposure time 96 h were used. The samples were randomly divided into five groups (n = 15): I—intact enamel (control group); II—artificial white spot lesion; III—artificial WSL treated with fluoride varnish; IV—artificial WSL treated with casein phosphopeptide—amorphous calcium phosphate (CPP-ACP) paste; V—resin-infiltrated artificial WSL. The surface microhardness was determined using the Oliver–Pharr method and a spherical indenter (Shimadzu Indenter, Kyoto, Japan). One-way analysis of variance (ANOVA) followed by a Post Hoc test (Bonferroni) was used with a level of significance set at *p* < 0.05. **Results:** Resin-infiltrated white spot lesions showed comparable microhardness mean value as the control group: 68.23 (±21.45) and 63.57 (±18.89), respectively (*p* > 0.05). Also, resin infiltration increased enamel microhardness compared to WSL values, with a statistically significant difference (*p* < 0.05). Fluoride varnish and CPP-ACP treatment resulted in equivalent values (50.84 ± 14.35 and 50.99 ± 15.31, respectively). **Conclusions:** Different WSL treatments (fluoride varnish, CPP-ACP and resin infiltration) produced comparable enamel microhardness values. Among the tested agents, resin infiltration resulted in higher microhardness values, while fluoride varnish and CPP-ACP demonstrated equivalent outcomes.

## 1. Introduction

Dental caries is a multifactorial, noncommunicable oral disease that is highly dependent on nutritional habits [1]. The contemporary understanding of caries etiology involves a combination of biological, behavioral, psychological and environmental factors. According to the Global Burden of Diseases, Injuries, and Risk Factors Study, dental caries is recognized as a major public health concern. It is estimated that 2.5 billion people suffer from caries of permanent teeth, and more than 530 million children suffer from untreated caries in primary dentition [2,3,4].

An initial enamel caries lesion, commonly referred to as a white spot lesion (WSL), is the first clinical sign of dental caries. Demineralization within the lesion, which occurs in the subsurface enamel layer, increases the porosity and modifies the optical properties of the enamel. The refractive indices of enamel, water and air differ, which causes the initial enamel caries lesion to appear as an opaque white spot, clearly distinguishable from intact enamel [4,5].

If this lesion is not detected in the initial phase and managed properly, it progresses to a cavity. Also, these opaque lesions might compromise the aesthetics of the patient if they are present in the aesthetic zone [6]. The conducted studies showed that WSLs on the anterior permanent teeth have a negative psychological impact among children and adolescents and that they lower self-esteem [7]. On the other hand, successful WSL management improves their oral health and quality of life [8]. Over the last decades, the prevalence of WSLs has increased [6,7].

Modern dentistry includes several non-invasive techniques in the treatment of white spot lesions. The aim is to arrest caries lesions at an early stage and to enhance their remineralization [9,10,11]. Fluoride-mediated remineralization is the cornerstone of caries prevention, and it is the most used concept worldwide [12]. Also, bioactive remineralizing agents such as amorphous calcium phosphate (ACP), casein phosphopeptide–amorphous calcium phosphate (CPP-ACP) and calcium silicate-based materials are commonly used [13,14,15]. CPP-ACP is a nanocomplex of calcium ions, phosphate ions and hydroxide ions stabilized by casein phosphopeptides. When pH values decrease, the CPP-ACP nanocomplex releases calcium and phosphate ions onto the tooth surface. This process enhances remineralization while simultaneously reducing demineralization. Additionally, a synergistic effect with fluoride has been reported, further contributing to the remineralization [12].

Micro-invasive approach in the WSL treatment includes infiltration of these lesions with a low viscosity composite resin. This resin, commercially available as ICON, infiltrates the body of the initial enamel caries lesion due to capillary forces and conceals the pores between the pathological crystals. Therefore, it creates a diffusion barrier inside the caries lesion body, and dissolution of crystals and caries progression are retarded. In addition, microporosities filled with resin provide a reflection of light similar to the surrounding intact enamel (RI of sound enamel is 1.62 and RI of resin infiltrant is 1.46) [13].

Non-invasive and minimally invasive strategies for caries management are widely employed. These approaches involve chemically distinct agents with different mechanisms of action and treatment protocols. Fluoride and casein phosphopeptide–amorphous calcium phosphate promote remineralization, while resin infiltration works by blocking diffusion pathways, thereby arresting caries progression. These agents are accessible and commonly used in clinical practice. Despite their differing modes of action, their effectiveness is often compared in the literature [14,15,16]. Some studies have also examined the impact of applying remineralizing agents prior to resin infiltration in the treatment of initial enamel caries lesions [17].

The obtainable clinical evidence reveals successful masking of enamel whitish discoloration in WSLs infiltrated with resin. However, there is further need for evaluation of its impact on other enamel features (such as surface roughness and enamel hardness, shear bond strength, penetration depth) [5]. In vitro studies using resin infiltration of artificial white spot lesions are a possible method to accomplish this goal [18].

Therefore, the objective of the present study was to evaluate the effect of different white spot lesion treatments on enamel surface microhardness.

The null hypothesis tested was that different white spot lesion treatments showed no significant difference in enamel surface microhardness values.

## 2. Materials and Methods

The present in vitro study was conducted in the Clinic for Pediatric and Preventive Dentistry and Department for Biochemistry, School of Dental Medicine, University of Belgrade, and Faculty of Technology and Metallurgy, University of Belgrade. Ethical Committee approval was obtained for the study protocol (No. 36/11; approved date: 16 April 2024).

### 2.1. Sample Collection

Seventy-five intact human premolars were used for the study. The inclusion criteria were intact maxillary or mandibular, and first or second premolars extracted upon orthodontic indication. The exclusion criteria were hypomineralization, hypoplasia, caries, restorations or fractures of the teeth. The patients and their parents/guardians received information about the study, and their written consent was obtained.

### 2.2. Specimen Preparation

The selected teeth were cleaned with periodontal curettes to remove any remaining soft tissue and with fluoride-free prophylactic paste (Cleanic, Kerr, Brea, CA, USA), applied by a brush mounted on a low-speed hand piece, under water cooling. In addition, the vestibular surfaces were polished using cups mounted on a low-speed hand piece, under water cooling. The teeth were washed in distilled water and stored in a 0.1% thymol solution at room temperature until further use for no longer than 1 month to prevent bacterial growth.

Roots were removed at the cementoenamel junction with a low-speed diamond saw (Isomet, Buehler Ltd., Lake Bluff, IL, USA) under water cooling. Pulp tissue was discarded, and pulp chambers of the premolar crowns were obturated with flowable composite (Revolution Flowable Composite, Kerr, Brea, CA, USA). The premolar crowns were divided into five groups using simple randomization (one control group and four test groups; n = 15):

I—Intact enamel (control group);
II—Artificial white spot lesion (WSL);
III—Artificial WSL treated with fluoride varnish;
IV—Artificial WSL treated with CPP-ACP paste;
V—Resin-infiltrated artificial WSL.

**Figure 1:**
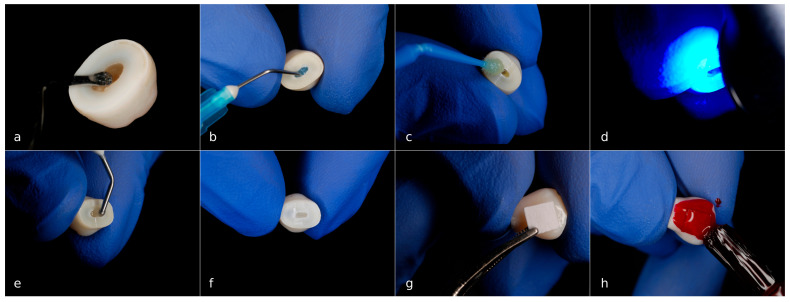
Preparation of the premolar crowns for the demineralization: (**a**) pulp tissue was removed; (**b**) dentin was etched with 37% orthophosphoric acid; (**c**) after rinsing and drying, an adhesive was applied; (**d**) polymerization was performed; (**e**,**f**) Pulp chambers were obturated with flowable composite; (**g**) the middle area of buccal surface was isolated using adhesive paper, sized 4 mm × 4 mm; (**h**) the crowns were coated with an acid-resistant nail varnish.

All crowns in test groups were coated with an acid-resistant nail varnish (Essence, Cosnova GmbH, Sulzbach, Germany), except for a middle area of vestibular surfaces, which were isolated using adhesive paper, sized 4 mm × 4 mm (Figure 1). The adhesive papers were removed after the nail varnish had dried completely at room temperature.

### 2.3. Artificial Demineralization

The results of previously conducted research showed that a demineralizing solution composed of acetic acid, monopotassium phosphate and calcium chloride with pH = 4.4 and exposure time 96 h produced optimal artificial WSLs [19]. Each specimen was submerged in 20 mL of the demineralizing solution in a sterile plastic container. The pH of the solution was checked every day with a probe to maintain the initially defined value (Mi 150, pH/Temperature Bench Meter, Martini instruments—Milwaukee Instruments, Rocky Mount, NC, USA). If necessary, it was adjusted with potassium hydroxide.

After demineralization, nail varnish was carefully removed with oil-free acetone, and the samples were thoroughly washed with distilled water. All specimens were visually inspected, and the macroscopic appearance of the enamel lesion was evaluated.

### 2.4. Material Application

The artificial initial enamel caries lesions were treated with three different agents whose composition is shown in Table 1. The specimens in groups I and II were stored in deionized water at room temperature.

Group III—Fluoride varnish

The group III samples were treated with Fluor Protector S (Ivoclar Vivadent, Schaan, Liechtenstein). The samples were dried with an oil-free air spray, and the varnish was applied with the brush (Vivabrush G, Ivoclar Vivadent, Schaan, Liechtenstein). After 60 s, each sample was immersed in deionized water at room temperature for 28 days.

Group IV—CPP-ACP paste

In this test group, Tooth Mousse (GC, Tokyo, Japan) was used. After drying with oil-free air spray, the WSLs were coated with paste for 5 min and were stored in deionized water in sterile plastic containers at room temperature. The remineralizing procedure was repeated for 28 days.

Group V—Resin infiltration

The artificial WSLs were treated with resin infiltrant—ICON (DMG, Hamburg, Germany) following the manufacturer’s instructions (Appendix A.1—Table A1). After resin application, the specimens were polished using cups on a low-speed hand piece under water cooling. The specimens were stored in deionized water in sterile plastic containers at room temperature for 28 days.

### 2.5. Surface Microhardness Testing

Silicone molds sized 1 × 1 cm were made. The samples (vestibular halves of premolar crowns) were embedded in acrylic blocks, leaving the vestibular surface of the crown exposed, flat, and as parallel as possible to the floor. The surface microhardness was determined using the Oliver–Pharr method and a spherical indenter (Shimadzu Indenter, Kyoto, Japan). The Oliver–Pharr method is an analytical technique used in instrumented indentation testing to evaluate the hardness of a material/object. To obtain the hardness value, mathematical analysis is performed [20,21]:

H = Pmax/A

In this formula: H—hardness; Pmax—peak load during the indentation; A—effective contact area between the indenter and the tested surface under maximum load.

**Figure 2:**
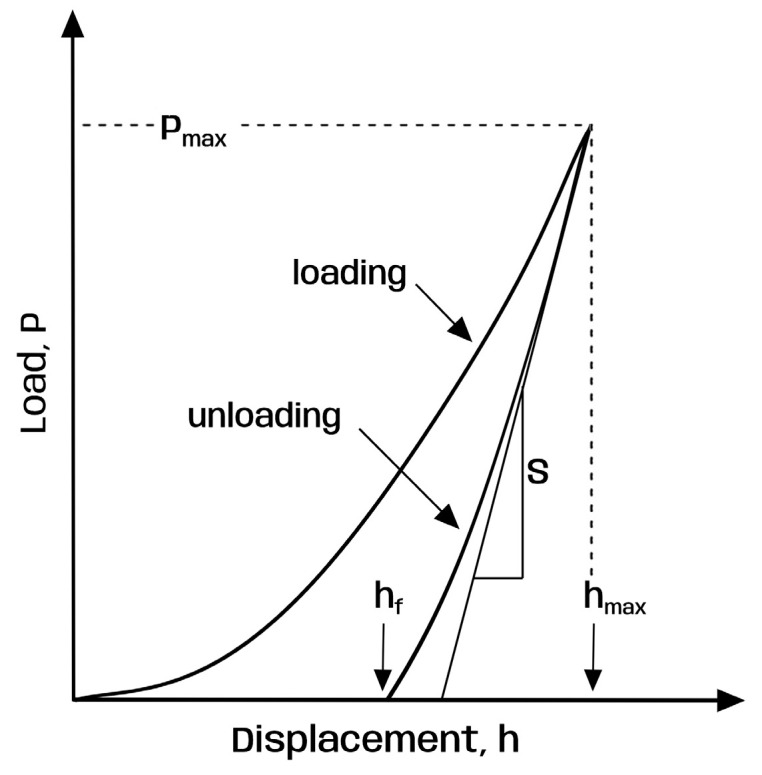
Schematic illustration of indentation load–displacement data showing important parameters for hardness analysis [20,21]: P—load, h, h_f_—displacement, S—the slope of the unloading curve at the beginning.

The variable A is calculated using the following formula:

A = 24.5⋅h_c_^2^

(h_c_—the depth of contact: h_c_ = h_max_ − ϵ⋅P_max_/S: h_max_—the largest penetration depth; ϵ—constant which depends on the type of the indenter; S—the slope of the unloading curve at the beginning; Figure 2).

**Figure 3:**
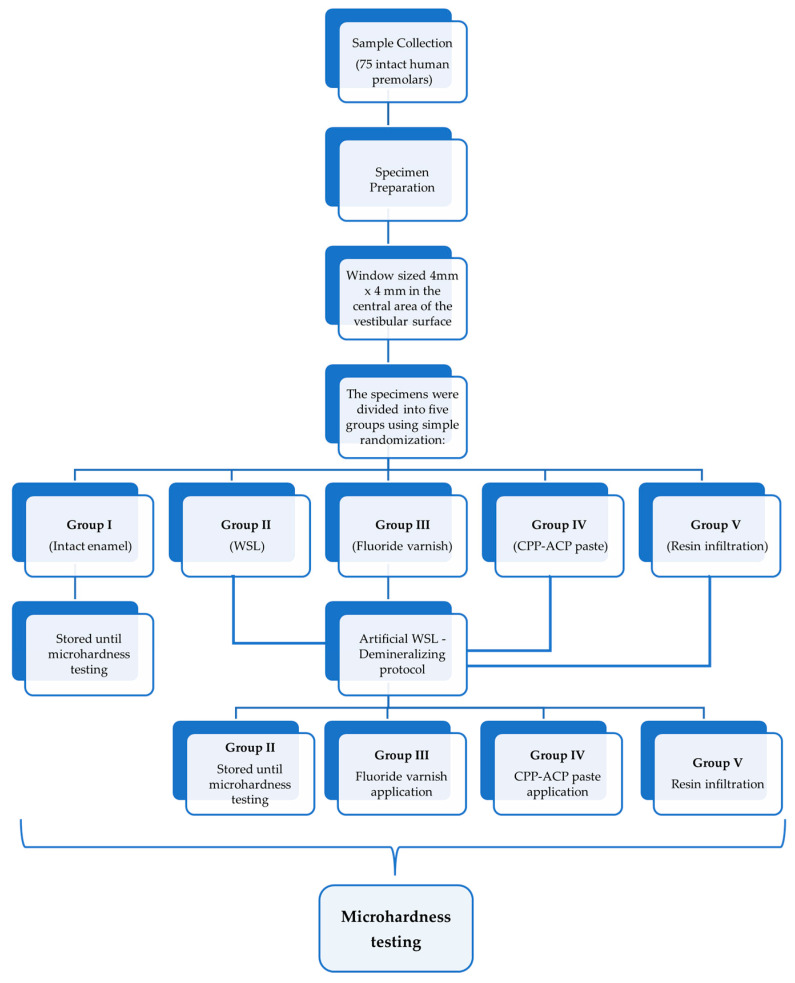
Flow chart of the sample collection and specimen preparation for microhardness testing.

**Figure A1:**
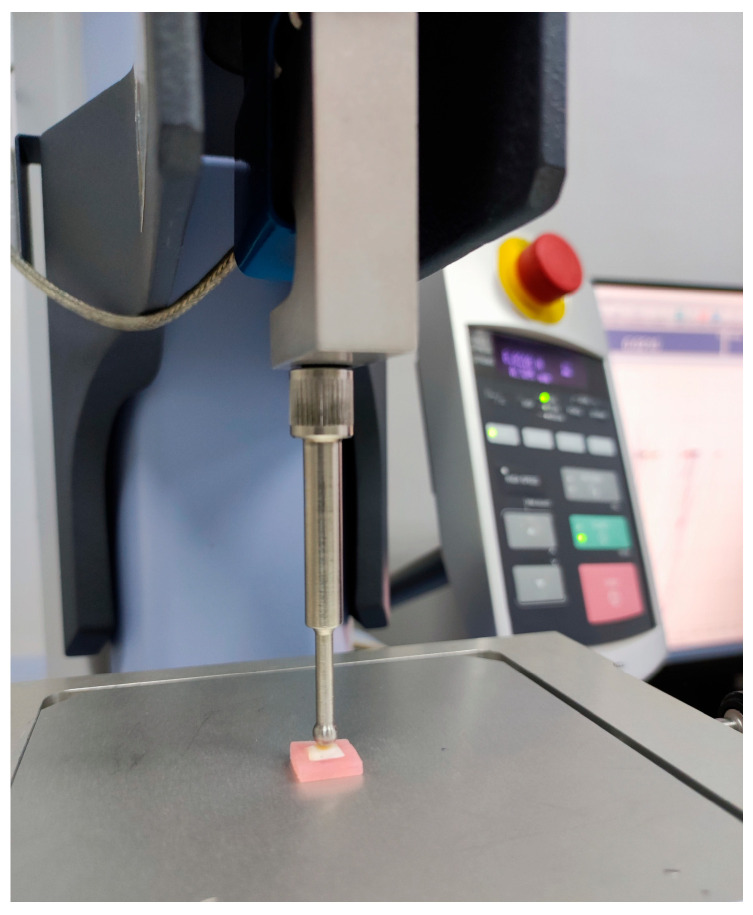
Surface microhardness testing.

Three indentations were made at the center of each sample (Appendix A.2—Figure A1), and the microhardness values were calculated according to the previously described mathematical model. The average value for each sample was calculated. The flow chart of the sample collection and specimen preparation for microhardness testing is presented in Figure 3.

### 2.6. Statistical Analysis

SPSS version 22.0 (IBM Corp., New York, NY, USA) was used for the statistical analysis of the data obtained. The microhardness results were analyzed by one-way analysis of variance (ANOVA) followed by Post Hoc multiple comparisons (Bonferroni test). The level of significance was set at *p* < 0.05.

## 3. Results

**Figure 4:**
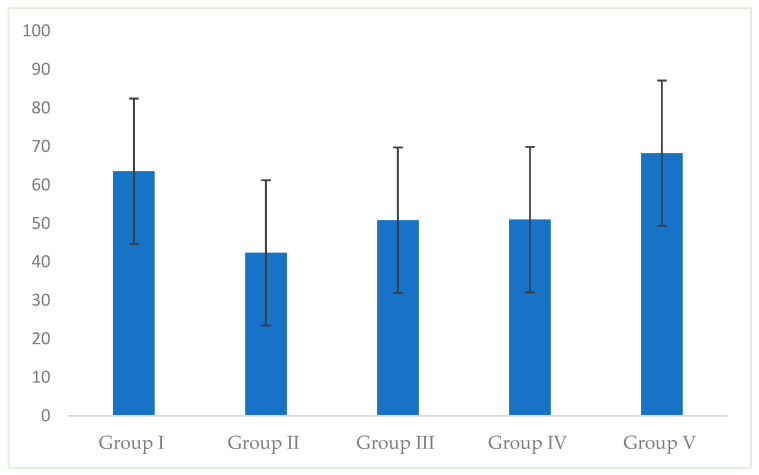
Mean enamel microhardness values (with standard deviation) in all groups.

The highest mean microhardness values were obtained in group I (intact enamel) and group V (resin-infiltrated WLSs). In group I mean value was 63.57 ± 18.89. Comparable results were found in group V, which ranged from 31.66 to 112.74 with a mean of 68.23 ± 21.45 (Figure 4, Table 2). Between these two groups, there were no statistically significant differences (*p* > 0.05).

Group III (fluoride varnish-treated WSLs) and group IV (CPP-ACP paste-treated WSLs) demonstrated equivalent results: mean values were 50.84 ± 14.35 and 50.99 ± 15.31, respectively. There was no statistically significant difference between groups III, IV and V (*p* > 0.05).

The lowest values of enamel surface microhardness were found in group II (WSLs). The mean value was 42.35 ± 17.98. There was a statistically significant difference between groups I and II and groups II and V (*p* < 0.05).

## 4. Discussion

Dental caries, as one of the most common oral diseases worldwide, represents a major public health concern [2]. Under physiological conditions, the enamel surface is subjected to continuous cycles of demineralization and remineralization. However, when pathological factors predominate, demineralization surpasses remineralization, leading to the formation of an initial enamel caries lesion [22]. This stage of caries development is highly dynamic and reversible. Advances in the understanding of the etiological factors and pathophysiological processes involved in dental caries have led to a paradigm shift in the approach to caries management. The minimally invasive concept has been introduced, emphasizing the crucial role of preventive and prophylactic therapy and early caries detection [23]. Also, individual caries risk assessment is a fundamental part of the treatment. Resin infiltration, as a micro-invasive approach, is part of the minimum intervention dentistry concept (MID) [24].

There are many demineralization protocols for artificial WSLs formation. They can contain lactic acid, acetic acid, methyl diphosphonate (MHDP), an acidified hydroxietilcellulose system, and in some of them, fluoride might be added. The results of the systematic review and meta-analysis showed that the duration of the demineralization process varied from several hours to 1.200 h. In addition, pH of demineralizing solutions varied between 4 and 5 [25]. In the present study, the demineralizing solution was selected due to the results obtained by a previously conducted pilot study [19].

Enamel microhardness evaluation requires the testing surface to be as flat and parallel to the base as possible. Some authors used silicon carbide discs under water cooling and polishing procedures to flatten the enamel [18,26,27]. Also, the removal of about 100 μm of the outer enamel layer was reported [18]. The authors of the present study wanted to preserve the top enamel layer and to avoid the flattening procedures. Therefore, the spherical micro-indenter and the Oliver–Pharr method were chosen.

The Oliver–Pharr method is an analytical technique used in indentation testing (microindentation or nanoindentation) to determine the hardness and elastic modulus of a material. It was developed by W.C. Oliver and G.M. Pharr in the 1990s, and it has been improved over the years for testing of micro/nano-structures, biomaterials and coatings [20,21]. The Vickers and Knoop hardness testing involve precise measurement of indenter impression [28]. In contrast, the Oliver–Pharr method enables direct determination of mechanical properties from the indentation load-unloading curve, eliminating the need to visually assess the hardness impression. Accurate indenter impressions are ideally obtained when the testing surface is flat; if this condition is not met, the measurement of hardness values becomes imprecise. Given that the enamel surface, without additional preparation, is not entirely flat or parallel to the base, the Oliver–Pharr method was selected as the more reliable option for the present study.

Enamel surface microhardness testing can reflect the improvements in WSLs after resin infiltration. This measurement cannot assess the mineral changes in the WSL after the different treatment methods, but it can demonstrate the improvements of the WSL [10,29,30]. The results of this study showed that resin infiltration of WSLs significantly increased the enamel surface microhardness compared to the untreated ones. Some of these measurements were equivalent to sound enamel. However, a statistically significant difference was not found between different white spot lesion treatments. So, the null hypothesis was accepted. The lowest mean microhardness was found in the white spot lesion group, which was expected and in accordance with the literature data [9,31,32]. It might be explained by chemical remodeling of the enamel rods, which caused pore formation and reduced microhardness [9].

The resin infiltrant organic matrix is mostly composed of triethlylene glycol dimethacrylate (TEGDMA). This monomer has a high degree of conversion, low viscosity and low molecular weight. Another benefit is the ability to penetrate the porous structure of the WSL body and to prevent further ionic diffusion [31]. The incorporated resin matrix might reinforce the enamel structure mechanically. The microhardness finding by Paris et al. also showed that resin infiltration can reharden the WSL [33]. Mandava et al. demonstrated better results of resin infiltration in recovering microhardness compared to colloidal silica infiltrates [10]. However, most of them reported lower microhardness in artificial WSLs treated with resin infiltrant compared with sound enamel [30,33]. This might be associated with the fact that the degree of conversion in some cases can be lower, and formation of chains might not always occur [34]. Moreover, material shrinkage during light curing may leave some parts of demineralized enamel without infiltrant, which could also contribute to the decrease in surface microhardness compared to intact enamel [30].

Fluoride remains the gold standard for white spot lesion treatment with multiple systematic reviews confirming its successful effects [25,26,27]. The literature suggests that a fluoride varnish is highly effective in reducing caries, providing a long-lasting effect with minimal side effects [35,36]. Additionally, it is well-tolerated by both patients (children and adults) and clinicians. In contrast, fluoride solutions, while less effective for long-term remineralization, offer a more economical alternative. In the present study, varnish containing organic fluoride was used. The initial fluoride concentration in it is 7700 ppm, which is lower compared to the conventionally used 5% sodium fluoride (NaF). However, according to the product information, after varnish application, the solvent evaporates, and the effective fluoride concentration is increased on the tooth surface. Moreover, organic fluoride forms a stable film on the tooth surface due to its surfactant-like properties, which enhance its retention time and contribute to prolonged remineralizing and antibacterial activity [37]. The organic fluoride varnish might be applied in a very thin layer that dries swiftly, which is convenient and more comfortable for the pediatric population. Therefore, this varnish was selected for the study.

CPP-ACP is one of the commonly used remineralizing agents and its efficacy has been investigated [38]. Scientific evidence supports the use of CPP-ACP for remineralization of early enamel caries lesions. However, the clinical benefit of CPP-ACP use over fluoride is still unclear [39]. It might be challenging to simulate CPP-ACP paste usage in the in vitro conditions. Some authors applied it once a day for 14 days [40], while Rana et al. reported applying it twice a day for 14 days [9]. A few studies described daily application for 28 days as a relevant treatment duration [41,42,43] and this recommendation was incorporated into the present study.

The findings of the present study demonstrated similar average enamel microhardness values between the fluoride varnish and the CPP-ACP paste groups. These values were lower than sound enamel but above the WSL microhardness. These results were in accordance with literature data [44]. The low-viscosity resin infiltrant penetrated early enamel caries lesions immediately, and therefore, the microhardness recovery can be measured after the treatment. In the oral cavity, the effects of fluoride varnish and CPP-ACP are saliva-dependent. Also, in the present study, the CPP-ACP paste was applied for 4 weeks. Despite the limitations of an in vitro study, both fluoride therapy and the CPP-ACP paste showed higher average enamel microhardness values compared to the average microhardness value of white spot lesions. However, the statistically significant difference was not found (*p* > 0.05). In further research, a prolonged period of use may be considered, and the results could be compared to the results obtained from the present study. The literature data suggested that fluoride varnish used in the present study showed a positive therapeutic effect and decreased demineralization [45,46]. On the other hand, an in vitro study that evaluated fluoride release 6 months after application demonstrated that 5% NaF was superior to ammonium fluoride-based varnishes [47]. In future research, the in vivo effects of these products can be examined.

In vitro studies are commonly employed to assess various enamel surface characteristics, including hardness, surface roughness and biofilm susceptibility. However, the oral cavity represents a dynamic environment characterized by pH fluctuations, mechanical forces and the presence of saliva, which plays a critical role in maintaining overall oral homeostasis. The present study is limited by its inability to account for saliva-dependent reactions and pH variations. Future research could investigate the impact of additional demineralization on treated enamel surfaces, mineral content changes (SEM/EDX) and long-term mechanical characteristics. Another important aspect of the treatments evaluated is their aesthetic outcome, which requires further investigation. In future research, an alternative approach could be explored, including measuring the initial microhardness of the enamel before demineralization, WSL microhardness and then various white spot lesion treatments. A preliminary microhardness test was not conducted in this study, and incorporating it into future studies would help validate the results. The results indicated no statistically significant differences among groups III, IV, and V. However, group V (resin infiltration) demonstrated outcomes comparable to the control group. It is possible that stronger statistical correlations could emerge with a larger sample size. This aspect warrants further investigation in future research.

## 5. Conclusions

The various treatments for white spot lesions (fluoride varnish, casein phosphopeptide–amorphous calcium phosphate and resin infiltration) produced comparable enamel microhardness values. Among the tested agents, resin infiltration resulted in higher microhardness values, while fluoride varnish and CPP-ACP demonstrated equivalent outcomes. Given the inherent limitations of this in vitro pilot study, further research is necessary to verify these findings, particularly under clinical conditions.

## Figures and Tables

**Table 1:** Composition of materials used in the study.

Material	Composition	Manufacturer
Fluor Protector S	1.5% ammonium fluoride (7700 ppmF in solution); Ethanol; Water; Polymer; Additive; Saccharin, mint aroma	Ivoclar Vivadent, Schaan, Liechtenstein
Tooth Mousse	CPP-ACP (RECALDENT), Water, Glycerol, D-sorbitol, CMC-Na, Propylene glycol, Silicon dioxide, Titanium dioxide, Xylitol, Phosphoric acid, Flavoring, Zinc oxide, Sodium-saccharin, Ethyl p-hydroxybenzoate, Magnesium oxide, Guar gum, Propyl p-hydroxybenzoate, Butyl p-hydroxybenzoate	GC, Tokyo, Japan
ICON	Icon-Etch: Hydrochloric acid (15% HCl), Pyrogenic silicic acid; Surface-active substances; Icon-Dry: 99% ethanol; Icon-Infiltrant: Methacrylate-based resin matrix; Initiators; Additives	DMG, Hamburg, Germany

**Table 2:** Microhardness values and comparison between groups.

	Group I Intact Enamel	Group II WSL	Group III Fluoride Varnish	Group IV CPP-ACP Paste	Group V Resin Infiltration
Mean	63.57 ^A^	42.35 ^B^	50.84 ^A,B^	50.99 ^A,B^	68.23 ^A^
S.D.	18.89	17.98	14.35	15.31	21.45
*p*	0.001 *				

*p*: One-way analysis of variance (ANOVA), * Significant difference at *p* < 0.05; Post Hoc multiple comparisons: Means with different superscripted letters were significantly different.

## Data Availability

The original contributions presented in this study are included in the article. Further inquiries can be directed to the corresponding author.

## Abbreviations

The following abbreviations are used in this manuscript:

WSL	White spot lesion
CPP-ACP	Casein phosphopeptide–amorphous calcium phosphate
RI	Refractive index
TEGDMA	Triethlylene glycol dimethacrylate
NaF	Sodium fluoride

## Appendix A

### Appendix A.1

**Table A1:** Protocol for resin infiltration (ICON) according to the manufacturer’s recommendations.

Steps	Recommendations
Step 1: Icon-etch	Apply Icon-etch with the tip; Let it set for 2 min with periodic smearing; Rinse with water for 30 s; Dry with oil/water-free air spray.
Step 2: Icon-dry	Apply Icon-dry with the tip; Let it set for 30 s; Dry with oil/water-free air spray.
Step 3: Icon-infiltrant	Apply Icon-infiltrant with the Icon Vestibular Tip; Let it set for 3 min with periodical activation and additional resin coating; Remove excess material with cotton rolls; Light cure for 40 s; Repeat the application of Icon-infiltrant, as previously described, with contact time 1 min; Polishing.
